# Dementias Platform UK and other similar platforms to advance research into the global epidemiology of dementia

**DOI:** 10.1002/alz.087348

**Published:** 2025-01-09

**Authors:** Sarah Bauermeister

**Affiliations:** ^1^ Dementias Platform UK ‐ University of Oxford, Oxford United Kingdom

## Abstract

**Background:**

Dementias Platform UK (DPUK) is a global data repository and analysis environment for optimising the use of existing longitudinal clinical and population data resources for dementias research. The DPUK Data Portal provides secure storage and access for high‐quality, multi‐modal data from over 65 cohorts across 3.6 million individuals. Data include clinical, survey, imaging and genetic outcomes and measures which allow for multi‐cohort comparison and validation work. Importantly, Data Portal resources are are free to access from anywhere in the world and targeting participation and collaboration with LMICs is a key focus. The remotely accesses data, mentoring and training resources provided by DPUK are enabling increasingly diverse international representation from LMIC researchers ‐ democratizing a research landscape traditionally more accessible for HIC researchers. Here we present the DPUK programme of work for democratizing data access and training for LMICs.

**Method:**

1. All bona fide academic and industry researchers may access the resources of the DPUK Data Portal.

2. Tools and guidance documents are provided for the identification of cohorts and data of interest.

3. An online application is completed and the median time for data owner response is 23 days.

4. Training workshops and academies are provided for researchers at very low cost or free to LMIC participants – providing online analytical and data management training.

5. A remote mentoring programme initiated between DPUK and the IHCC (International Health Cohorts Consortium) provides early career researchers (ECRs) with opportunity to work with senior researchers – especially beneficial for those in LMICs.

**Result:**

Over 428 research applications with 1038 users across 48 countries (11 LMICs) have been received by the Data Portal. More than 200 international researchers have attended training events across 12 countries (3 LMICs).

**Conclusion:**

DPUK is democratizing data access and training for all researchers, including those from LMICs where resources for research are difficult to acquire and bespoke training programmes are largely inaccessible. Only by making secondary data resources and training more easily accessible and affordable will it be possible to increase LMIC representation of researchers in dementia and neurodegeneration.

